# Diagnostic Complexity in Pediatric Chronic Colitis With Allergic, Infectious, Histamine-Related, and Oral-Phase Contributors: A Case Report With Long-Term Follow-Up

**DOI:** 10.7759/cureus.113273

**Published:** 2026-07-24

**Authors:** Gabriela N F Guimarães

**Affiliations:** 1 Independent Researcher, Nutrology, Porto Alegre, BRA

**Keywords:** case report, crohn-like colitis, histamine metabolism, microbiota, pediatric colitis

## Abstract

Pediatric chronic colitis may present with overlapping gastrointestinal, allergic, dietary, and microbiome-related features, making early therapeutic decisions challenging, particularly when immunosuppression is being considered. This report describes an eight-year-old boy with chronic diarrhea and intermittent hematochezia beginning in early childhood, a history of cow’s milk protein allergy, selective eating, poor mastication, inflammatory stool biomarkers, and colonoscopic findings consistent with mild chronic ileocolitis. Because the family declined systemic immunosuppression, a structured gut-focused approach was implemented over 18-24 months, including dietary elimination, reduction of dietary histamine burden, feeding therapy, digestive and diamine oxidase (DAO) support, microbiota modulation, and treatment of suspected enteric infections or overgrowth. Over time, the patient’s stool normalized, bleeding resolved, inflammatory markers improved substantially, and repeat colonoscopy demonstrated mucosal healing. This case highlights the potential relevance of individualized, nonimmunosuppressive gastrointestinal strategies in selected pediatric patients with chronic Crohn-like colitis and allergic or histamine-related features, while recognizing that causality cannot be established from a single case.

## Introduction

Early-onset inflammatory bowel disease (IBD) includes children who develop chronic intestinal inflammation during the first decade of life [1,2]. In this age group, phenotypes may overlap with food allergy, barrier dysfunction, and microbiota disturbances [3]. A subset of patients also exhibit features consistent with impaired histamine degradation capacity, which may contribute to gastrointestinal symptoms and mucosal immune activation [4-7].

In parallel, oral-phase dysfunction can be clinically relevant in selected children with chronic gastrointestinal complaints. Rapid swallowing, limited chewing, and restricted texture acceptance can increase delivery of larger food particles and incompletely processed macromolecules to the distal gut [7-10]. This may augment luminal antigen exposure and fermentation load and may worsen barrier stress in susceptible hosts [11].

We report a child with chronic colitis of Crohn-like phenotype and long-standing hematochezia who also had allergic features, impaired mastication/digestive processing, elevated stool histamine, and histamine-pathway genetic variants. The case describes a structured, nonimmunosuppressive, gut-focused multimodal approach and subsequent clinical and endoscopic evolution. This case report was prepared in accordance with the Case Report (CARE) reporting guideline, and a completed CARE checklist is provided as a supplementary material.

## Case presentation

An eight-year-old boy was referred for evaluation of chronic diarrhea and intermittent rectal bleeding that had been present since early infancy. His past medical history was notable for cow’s milk protein allergy diagnosed during infancy and orchidopexy for cryptorchidism at three months of age. Neurodevelopment was reported as normal, and toilet training was achieved at two years and two months. Family history was negative for IBD and positive for atopy.

From approximately two months of age, the child had recurrent blood-streaked stools, initially interpreted in the context of cow’s milk protein allergy. He remained exclusively breastfed until five months of age. Complementary feeding was introduced because of poor weight gain, but feeding difficulties persisted, with limited acceptance of textures and flavors. After weaning at two years of age, he transitioned to hypoallergenic formulas. Despite conventional allergy-directed dietary management, including avoidance of cow’s milk protein, he continued to have chronic loose stools, intermittent hematochezia, visible food fragments in stool, selective eating, and impaired chewing patterns.

At my first consultation with the patient, at approximately six years of age, the child passed two to three loose stools daily, sometimes associated with urgency and visible blood. Stools were described as foamy and disintegrating, with recognizable food fragments, including potato, corn, and fruits. Parents reported rapid swallowing, minimal chewing, and refusal of harder textures. There was little gas, no major abdominal distension, and episodic vomiting. He appeared thin for age but remained active. Dietary intake was heavily centered on refined carbohydrates and processed foods, including sandwiches, pizza, and baked goods, with limited fruit and vegetable acceptance.

The child lived with his family and had consistent parental involvement throughout the diagnostic and therapeutic process. The family’s main concern was the persistence of diarrhea and intermittent hematochezia despite long-standing dietary vigilance. Gastroenterological evaluation was pursued after colonoscopy described macroscopic findings compatible with IBD and suggestive of Crohn’s disease; however, histopathology showed mild chronic ileocolitis without granulomas or other definitive features sufficient to establish a diagnosis of Crohn’s disease. Because of concerns regarding long-term systemic corticosteroids, immunomodulators, and biologic therapy, the family declined systemic immunosuppressive treatment and elected close longitudinal monitoring with a structured, nonimmunosuppressive, gut-focused approach.

At the first clinical evaluation, the child weighed 22 kg and measured 122 cm in height, corresponding to a body mass index of 14.8 kg/m², approximately at the 30th-35th percentile for age and sex. He was clinically stable and afebrile. Abdominal examination was unremarkable, with no distension, focal tenderness, palpable masses, or signs of acute abdominal inflammation. Perianal examination was normal, without fissures, fistulas, abscesses, skin tags, or perianal ulceration. There were no oral aphthous ulcers and no extraintestinal manifestations suggestive of IBD, including arthritis, skin lesions, ocular inflammation, or unexplained fever. Orofacial myofunctional assessment identified a restrictive lingual frenulum with reduced tongue elevation and lateralization, consistent with impaired bolus manipulation and inefficient mastication.

Available growth and routine laboratory evaluations were within expected limits. C-reactive protein, erythrocyte sedimentation rate, albumin, hemoglobin, ferritin, vitamin D, vitamin B12/folate, and platelet count were within laboratory reference ranges, indicating no overt systemic inflammation, anemia, hypoalbuminemia, or clinically significant micronutrient deficiency at the time of assessment. Celiac disease was considered in the differential diagnosis; however, anti-tissue transglutaminase testing was normal, and upper gastrointestinal biopsies did not show histopathological features consistent with celiac disease.

Diagnostic assessment

Endoscopy and Histopathology

**Figure 1:**
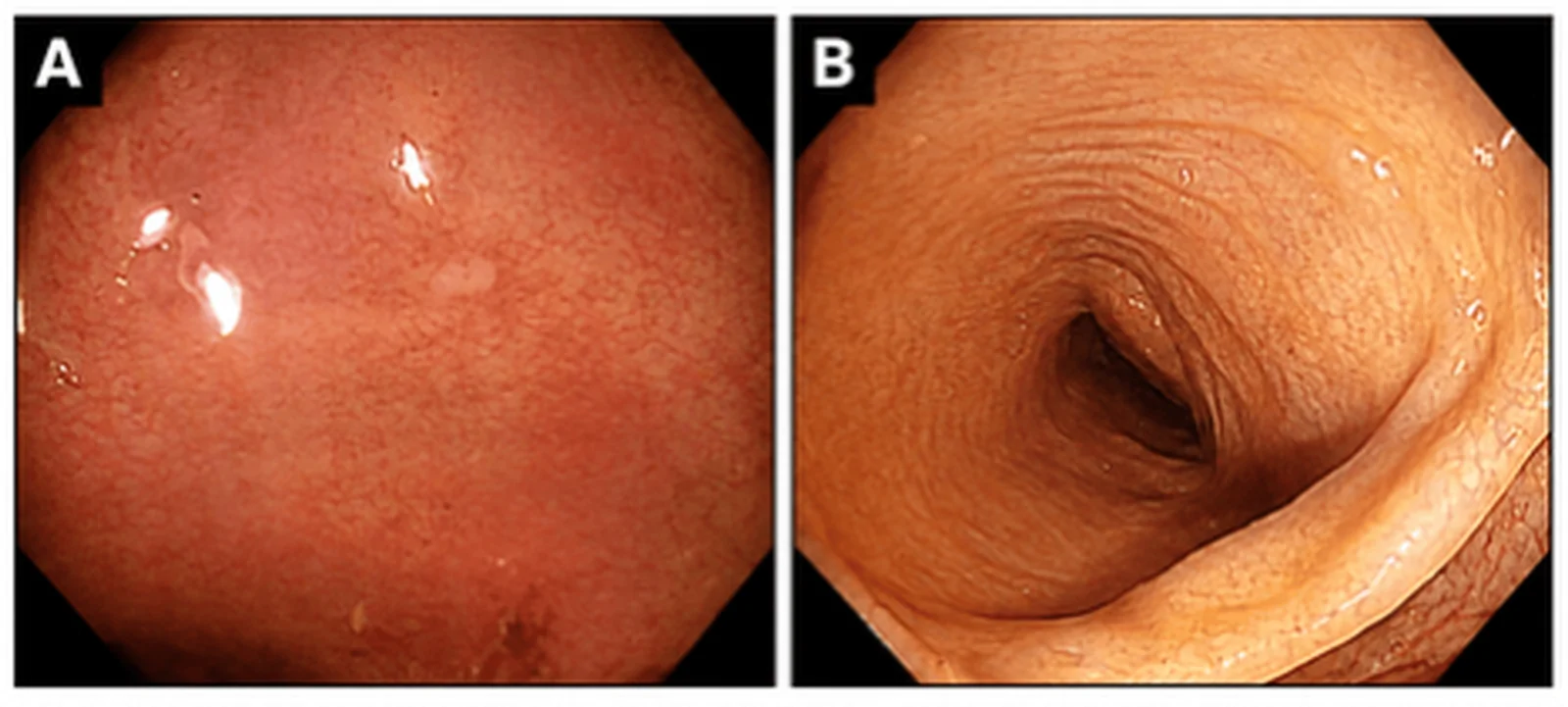
Colonoscopic appearance before and after treatment (A) Pretreatment image showing erythematous, granular mucosa with focal whitish punctate lesions suggestive of erosions or aphthoid ulcers and a reduced vascular pattern. (B) Posttreatment image showing endoscopic mucosal healing, with a smoother mucosal surface and no visible erosions in the field shown

At six years and three months, colonoscopy performed for suspected IBD demonstrated aphthoid ulcers in the rectum, sigmoid, descending, and transverse colon, with intervening normal mucosa (Figure 1); the cecum and ascending colon appeared normal. The terminal ileum showed mild erythema. The endoscopic impression was compatible with IBD of Crohn's disease phenotype.

Histopathology reported mild chronic ileitis and mild chronic colitis with lymphoid hyperplasia, with mild chronic nonspecific inflammation in rectal mucosa. There were no granulomas, no crypt abscesses, no cryptitis, and no architectural distortion. Upper gastrointestinal biopsies showed mild chronic gastritis without *Helicobacter pylori.*

Stool Inflammatory Markers, Digestive Profile, Microbiome, Infectious, and Histamine-Pathway Assessment

The rationale for the expanded diagnostic assessment was the coexistence of chronic diarrhea, intermittent hematochezia, prior allergic history, selective eating, poor mastication, visible undigested food fragments, and inflammatory stool biomarkers in a child with endoscopic findings suggestive of IBD. Because standard anatomical and histopathological evaluation did not fully explain the clinical complexity, additional stool, infectious, digestive, microbiome, and histamine-pathway assessments were pursued to characterize potentially modifiable contributors, including impaired digestion, altered intestinal barrier activity, microbial imbalance, occult infection, and histamine-related mechanisms.

A comprehensive stool, serum, microbiome, infectious, and histamine-pathway assessment was performed by Lemos Laboratory, Minas Gerais, Brazil, as part of the clinical diagnostic work-up. Fecal calprotectin, lactoferrin, zonulin, and secretory IgA were measured using automated enzyme immunoassay-based methods. Fecal histamine and fecal melatonin were quantified by liquid chromatography-mass spectrometry coupled to high-performance liquid chromatography. Qualitative and functional stool digestion analysis was performed using optical microscopy combined with an automated microscopy system. Fecal microbiota composition was assessed by 16S rRNA sequencing. Stool antigen testing for *Giardia *and *Clostridium *was performed using solid-phase chromatography. Histamine-pathway genetic testing, including amine oxidase copper-containing 1 (AOC1)/diamine oxidase (DAO), histamine N-methyltransferase (HNMT), monoamine oxidase B (MAOB), methylenetetrahydrofolate reductase (MTHFR), and aldehyde dehydrogenase 2 (ALDH2), was performed by real-time polymerase chain reaction. Serum DAO concentration and functional histamine degradation capacity were assessed using automated enzyme immunoassay-based methods.

The comprehensive stool panel obtained after colonoscopy showed increased inflammatory markers, including elevated fecal calprotectin and markedly elevated fecal lactoferrin (Table 1). Barrier-related markers were also increased, with elevated fecal zonulin and secretory immunoglobulin A (IgA). Fecal histamine was elevated, whereas fecal melatonin was reduced (Table 1). Qualitative digestive assessment identified undigested starch, digestible cellulose, and muscle fibers, consistent with impaired digestion and/or inadequate mastication.

**Table 1:** Stool biomarkers and qualitative digestive assessment results before and after treatment IgA: immunoglobulin A

Stool marker	Unit	Baseline (pretreatment)	After 18 months (posttreatment)	Reference interval
Fecal calprotectin	µg/g	318	54	<200
Fecal lactoferrin	µg/mL	28,106	8,427	<7,200
Fecal zonulin	µg/mL	153	130	<80
Fecal secretory IgA	mg/dL	722	5	51-204
Fecal histamine	µg/g	752	143	<500
Fecal melatonin	pg/mL	9	149	15-297
Qualitative digestive assessment	Qualitative	Undigested starch; digestible cellulose; muscle fibers	Some undigested muscle fibers	Not applicable

Fecal 16S rRNA microbiome analysis showed reduced diversity and depletion of genera commonly considered beneficial or barrier-supportive, including *Akkermansia*, *Faecalibacterium*, and *Bifidobacterium*, with overrepresentation of taxa often associated with inflammatory or dysbiotic intestinal states. Stool antigen testing for *Giardia *and *Clostridium* was positive. Because these findings were identified and treated during the multimodal intervention sequence, infection- and overgrowth-directed therapy represents an important confounder and may have contributed to the observed clinical and biomarker improvement.

Targeted single nucleotide polymorphism (SNP) analysis of histamine- and methylation-related pathways identified selected variants of potential functional relevance, including heterozygous variants in AOC1/DAO, HNMT, and MTHFR, as well as a reported MAOB variant interpreted according to the laboratory report. These findings were considered susceptibility-related context involving histamine degradation, methylation, and biogenic amine handling, rather than diagnostic or causal markers. They were interpreted alongside the child’s allergic history, elevated fecal histamine, dietary pattern, impaired oral-phase processing, and Crohn-like inflammatory findings.

Serum DAO concentration was in the low-normal range. A separate functional histamine degradation assay was interpreted as showing reduced histamine degradation capacity.

Timeline Summary

**Figure 2:**
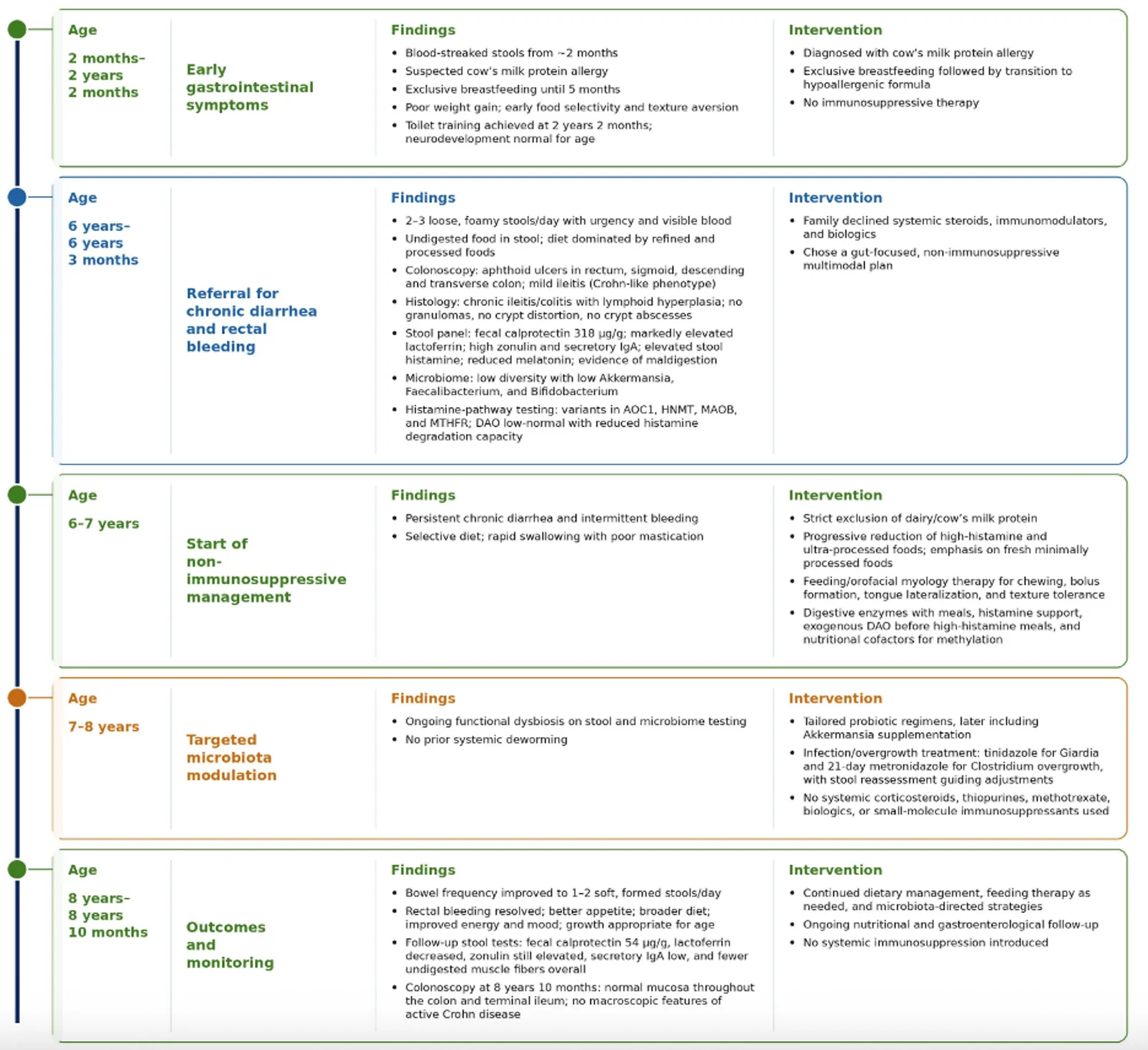
Clinical timeline, key findings, and nonimmunosuppressive management Image credit: created by the author using BioRender

Symptoms began in early infancy with hematochezia and suspected cow’s milk protein allergy. Chronic loose stools and intermittent bleeding persisted into early childhood. At age six years and three months, colonoscopy and histology supported a Crohn-like inflammatory phenotype. A gut-focused multimodal protocol was implemented without systemic immunosuppression and continued over approximately 18-24 months. At age eight years, symptoms and stool inflammatory markers had improved substantially, and at age eight years and 10 months, repeat colonoscopy showed near-complete mucosal healing (Figure 2).

Diagnostic challenges

Diagnostic challenges included the clinical overlap between pediatric IBD (PIBD), chronic allergic or food-sensitive colitis, infectious/parasitic colitis, histamine-related symptom amplification, microbiota disruption, and oral-phase digestive dysfunction. The absence of granulomas, crypt abscesses, cryptitis, architectural distortion, perianal disease, and extraintestinal manifestations made definitive classification as classic Crohn's disease less certain at baseline. In addition, no systematic monogenic IBD or immunologic workup was available in the clinical records, which limited etiologic stratification. No formal pediatric disease activity index or prognostic staging score was available retrospectively; therefore, prognosis was assessed longitudinally using clinical course, growth trajectory, fecal inflammatory biomarkers, and follow-up colonoscopy.

Diagnostic Considerations and Differential Diagnosis

The clinical phenotype and initial endoscopic findings raised concern for PIBD, particularly colonic-predominant Crohn's disease or IBD-unclassified, given the chronic diarrhea, hematochezia, segmental aphthoid ulceration, mild terminal ileal erythema, mild chronic ileitis/colitis, and elevated fecal inflammatory biomarkers. However, several findings argued against a classic, clearly progressive Crohn's disease phenotype at that time, including the absence of granulomas, crypt abscesses, cryptitis, architectural distortion, perianal disease, oral aphthous ulcers, unexplained fever, or extraintestinal manifestations.

The differential diagnosis also included chronic allergic or food-sensitive colitis, given the history of cow’s milk protein allergy, family history of atopy, selective diet, and possible food-related immune activation. Infectious or parasitic colitis was also considered because stool antigen testing was positive for *Giardia *and *Clostridium*, which informed subsequent infection- and overgrowth-directed treatment. Eosinophilic gastrointestinal disease was considered within the broader differential diagnosis; however, the available histopathology did not describe eosinophil-predominant inflammation. Celiac disease was considered less likely based on negative serologic testing and the absence of supportive histopathologic findings in the available records. Inborn errors of immunity or monogenic IBD were also considered as part of the broader differential diagnosis for early-onset intestinal inflammation; however, no systematic immunologic or monogenic IBD work-up was available in the clinical records.

Taken together, the available clinical, endoscopic, histologic, and fecal inflammatory biomarker findings supported a Crohn-like chronic inflammatory colitis within the PIBD spectrum. However, the absence of granulomas, crypt abscesses, cryptitis, architectural distortion, perianal disease, oral aphthous ulcers, unexplained fever, and extraintestinal manifestations made definitive classification as classic Crohn's disease less certain at baseline. Therefore, the case was interpreted conservatively as an IBD-spectrum/Crohn-like inflammatory phenotype with overlapping allergic, infectious/overgrowth, histamine-related, microbiota-related, and oral-phase digestive contributors, requiring longitudinal clinical, biomarker, and endoscopic follow-up.

Therapeutic intervention

The family expressed reluctance to long-term systemic corticosteroids, immunomodulators, or biologic therapy. A stepwise nonimmunosuppressive, gut-focused multimodal plan was implemented and adjusted according to clinical response, stool findings, and tolerability.

Dietary and Allergen-Load Interventions

Dietary management was individualized under nutritional supervision. Cow’s milk protein and foods identified as poorly tolerated or associated with hypersensitivity were excluded. Additional restrictions included selected highly fermentable foods, foods rich in antinutritional compounds such as phytates, oxalates, and tannins, and dietary components considered potential intestinal triggers, including gluten, casein, added sugars, ultra-processed foods, artificial colorants, preservatives, and monosodium glutamate. A strict low-histamine diet was followed for approximately 2 months, after which the intervention shifted to reduction of total dietary histamine load. Nutritional adequacy was supported through the progressive introduction of tolerated foods, including acacia gum, hydrolyzed beef protein, sources of mono- and polyunsaturated fatty acids, and antioxidant-rich foods. Food preparation and cooking methods were adapted to enhance digestibility and texture tolerance.

Mastication and Oral-Phase Feeding Therapy

**Figure 3:**
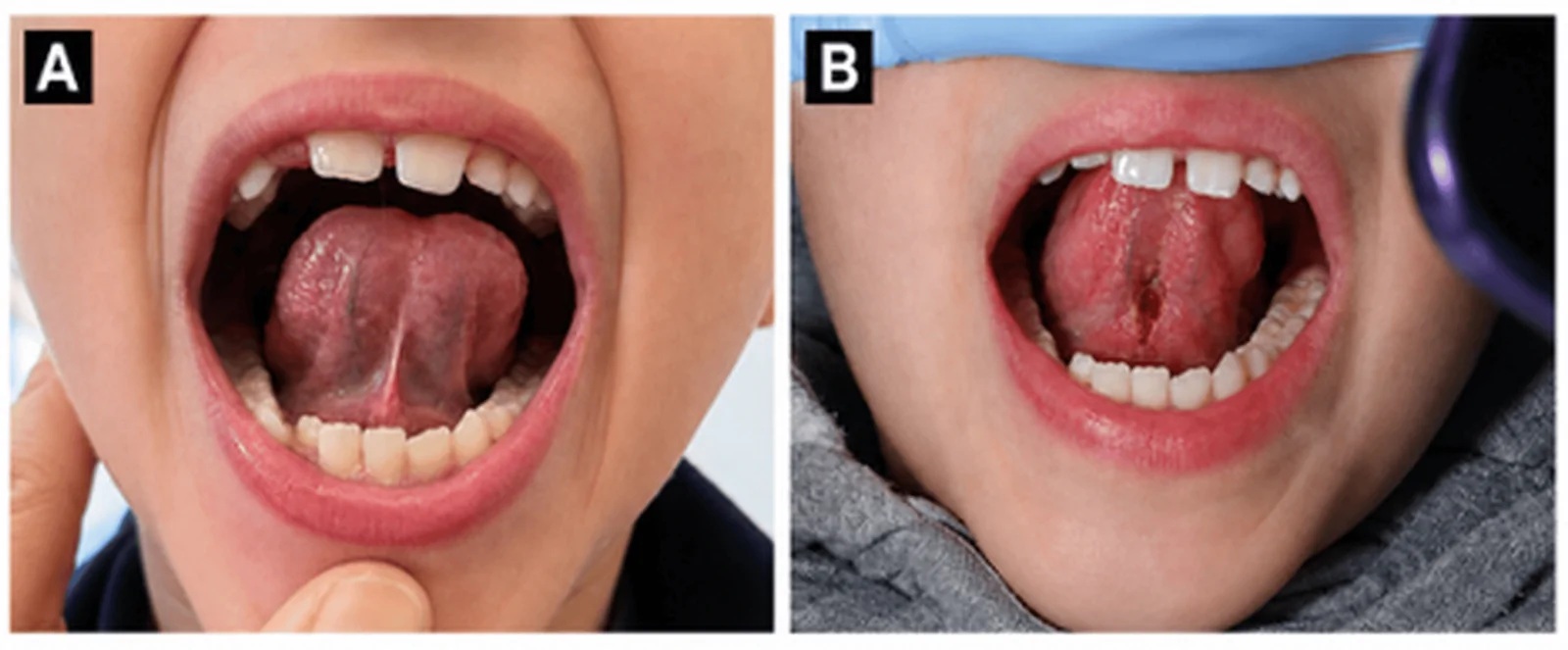
Pre- and postfrenectomy intraoral findings (A) Preoperative intraoral image showing a restrictive lingual frenulum with visible anterior tethering and reduced tongue elevation. (B) Postoperative intraoral image following lingual frenectomy, showing release of the tethered tissue and the immediate postoperative healing area

The child was referred for feeding therapy twice monthly and orofacial myology/speech therapy twice weekly for 8 weeks, targeting chewing efficiency, bolus formation, tongue lateralization, and progressive expansion of texture tolerance. During the therapeutic course, a restrictive lingual frenulum was identified as a potential anatomic contributor to reduced tongue mobility, impaired bolus manipulation, and inefficient mastication. Lingual frenectomy was performed in approximately March 2025 (Figure 3), followed by continued orofacial myology and feeding therapy focused on tongue mobility, chewing efficiency, bolus formation, and texture progression.

Digestive Support and Histamine-Load Mitigation

Pancrelipase was used as digestive enzyme support based on qualitative stool evidence of maldigestion and visible undigested food fragments, without implying a diagnosis of pancreatic exocrine insufficiency unless supported by pancreatic function testing. Pancrelipase delayed-release capsules (Creon 10,000) were administered with each main meal. Exogenous DAO supplementation was used before main meals at a dose of 2.1 mg, up to three times daily, particularly when meals were expected to carry a higher histamine load. Nutritional cofactor support for methylation and homocysteine metabolism was provided with a commercially available methylation-support supplement containing riboflavin-5′-phosphate 25 mg, pyridoxal-5′-phosphate 15 mg, 5-methyltetrahydrofolate 800 µg, vitamin B12 as methylcobalamin/adenosylcobalamin 1000 µg, and trimethylglycine 700 mg per capsule. This was used as supportive nutritional therapy in the context of the patient’s histamine- and methylation-pathway variants, without implying that the variants were diagnostic or directly causal.

Microbiota Modulation and Infection/Overgrowth Treatment

Based on stool and microbiome findings, targeted probiotic therapy was implemented, including a commercial *Lactobacillus rhamnosus* GG preparation (Culturelle). *Akkermansia muciniphila* supplementation (1 billion TFU) was later added for 2 months, given the baseline depletion of beneficial taxa. There was no prior systematic deworming early in the course. For a 22-kg child, nitazoxanide 200 mg twice daily with food for 3 days was prescribed for Giardia infection, followed by metronidazole suspension 40 mg/kg/day divided three times daily for 21 days, targeting suspected Clostridium-associated overgrowth based on stool antigen findings and clinical context. Stool reassessments and clinical response informed subsequent adjustments.

No systemic corticosteroids, thiopurines, methotrexate, biologics, or small-molecule immunosuppressants were used during follow-up. A summary of the therapeutic sequence, including the main interventions, rationale, and approximate timing, is provided in Table 3.

**Table 2:** Therapeutic sequence, rationale, and approximate timing of the nonimmunosuppressive gut-focused intervention AFU: active treatment units; CFU: colony-forming units; DAO: diamine oxidase The *Akkermansia muciniphila* AKK1 preparation was reported with strain deposit number CCTCC M 2023117

Period	Intervention	Rationale	Dose/frequency/duration
Baseline/early phase	Dairy/cow’s milk protein exclusion	Prior cow’s milk protein allergy and possible food-sensitive colitis	Continuous during follow-up
Early phase	Reduction of high-histamine and ultra-processed foods	To reduce dietary histaminergic amine load and processed-food exposure	Progressive implementation under nutritional supervision
Early phase	Pancrelipase	Visible food fragments and qualitative stool evidence of maldigestion	Pancrelipase delayed-release capsules, Creon 10,000, with main meals
Early phase	DAO supplementation	Elevated fecal histamine and reduced functional histamine degradation capacity	2.1 mg before main meals, up to three times daily
Microbiota phase	*Lactobacillus rhamnosus* GG	Microbiota modulation	5 × 10⁹ CFU daily; continued during follow-up
Microbiota phase	*Akkermansia muciniphila *AKK1	Baseline depletion of beneficial taxa, including *Akkermansia*	1 × 10⁹ AFU daily for 2 months
Infection/overgrowth phase	Nitazoxanide	Positive stool antigen testing for *Giardia*	200 mg twice daily with food for 3 days
Infection/overgrowth phase	Metronidazole	Suspected *Clostridium*-related overgrowth signal based on stool testing and clinical context	40 mg/kg/day divided three times daily for 21 days
Oral-phase intervention	Feeding therapy and orofacial myology	Inefficient mastication, impaired bolus formation, and limited tongue mobility	Feeding therapy twice monthly; orofacial myology twice weekly for 8 weeks
Surgical/oral-motor phase	Lingual frenectomy	Restrictive lingual frenulum with impaired tongue mobility and bolus manipulation	Performed in March 2025; followed by continued orofacial myology and feeding therapy

Follow-up and outcomes

Clinical improvement occurred progressively rather than immediately after a single intervention. Early management focused on dairy exclusion, reduction of high-histamine and ultra-processed foods, digestive enzyme support, DAO supplementation, and probiotic therapy. Persistent oral-phase dysfunction and visible food fragments in stool prompted structured feeding and orofacial myology therapy, followed by lingual frenectomy in March 2025. Over the subsequent months, the parents reported greater chewing efficiency, broader texture acceptance, reduced stool urgency, and gradual resolution of visible rectal bleeding. Because the intervention was multimodal and sequential, the relative contribution of each component cannot be determined.

Over approximately 18-24 months, bowel frequency decreased to 1-2 formed or softly formed stools per day, and visible blood resolved. Parents reported reduced urgency, improved appetite, broader food repertoire, and better energy and mood. Growth trajectory was reported as appropriate, without weight loss.

Repeat stool testing at age eight years demonstrated substantial improvement in inflammatory and histamine-related markers compared with baseline. Fecal calprotectin decreased to a near-normal range, and fecal lactoferrin decreased markedly, although it remained elevated. Fecal zonulin remained elevated, suggesting persistent barrier-related abnormalities, while secretory IgA decreased to a low level. Fecal histamine normalized, and fecal melatonin also returned to the expected range. Some undigested muscle fibers persisted on qualitative digestive assessment; however, the overall inflammatory, histamine-related, and digestive profile showed meaningful improvement compared with baseline (Table 1).

A second colonoscopy at age eight years and 10 months performed for monitoring showed normal mucosa throughout the colon and terminal ileum (Figure 1). No macroscopic features of active Crohn's disease were reported. The patient remained off systemic immunosuppression and continued nutritional and gastroenterological follow-up.

Adherence, tolerability, and adverse events

Adherence was assessed through serial clinical follow-up, parental reports, review of dietary implementation, and monitoring of symptom evolution during the therapeutic sequence. The family reported consistent adherence to dairy exclusion, progressive reduction of high-histamine and ultra-processed foods, digestive support, microbiota-directed supplementation, and feeding/orofacial therapy. No serious adverse events were reported during the follow-up period. No complications related to lingual frenectomy were documented in the available clinical records. Transient gastrointestinal discomfort related to dietary changes, supplementation, or antimicrobial therapy was not reported as clinically significant.

## Discussion

This case is discussed across three axes: (1) Crohn-like phenotype and the histamine-diet axis; (2) barrier, oral-phase dysfunction, and digestion as amplifiers of mucosal stress; and (3) remission without immunosuppressants under a multimodal intestinal strategy.

Crohn-like phenotype and the histamine-diet axis

This case presented with a phenotype compatible with PIBD: chronic diarrhea with hematochezia, segmental aphthoid ulceration on ileocolonoscopy, and markedly elevated fecal inflammatory biomarkers. In children, current diagnostic frameworks emphasize comprehensive endoscopic assessment with systematic biopsies and integration of fecal inflammatory markers (e.g., calprotectin, lactoferrin), which are highly sensitive for mucosal inflammation but not specific for IBD subtyping [12,13]. Accordingly, an “IBD spectrum” interpretation, including IBD-unclassified (IBD-U) or colonic-predominant Crohn-like disease, often requires longitudinal correlation across clinical course, endoscopy, histology, and objective biomarkers rather than a single snapshot [1,2,14].

Within this inflammatory colitis framework, histamine biology provides a plausible modifier axis rather than a stand-alone diagnosis. Histamine is produced endogenously (notably by mast cells and other immune cells) and can also derive from diet and microbial metabolism; its receptors are expressed in the gut and can modulate epithelial and immune responses [6,15]. Contemporary reviews summarize multiple points of crosstalk between histamine signaling and IBD-relevant immune pathways (including receptor-specific pro- vs. anti-inflammatory effects), but the field remains mechanistically complex and not yet sufficiently mature for routine clinical phenotyping based on histamine alone [16].

From a translational standpoint, variation in histamine degradation capacity (e.g., via DAO and HNMT pathways) and diet-related biogenic amine exposure may influence symptom expression (diarrhea urgency, abdominal pain, flushing-like symptoms) in selected patients, particularly when barrier vulnerability is suspected. However, “histamine intolerance” remains diagnostically challenging and lacks universally accepted biomarkers; serum DAO has been explored as a correlate of mucosal injury in some contexts but is not a definitive diagnostic tool. In this case, the combination of allergy history, fecal histamine findings, and pathway-related genetic variants is best framed as supportive context for a histamine [5,8,17,18], diet hypothesis that could modulate mucosal immune tone, while maintaining objective inflammation monitoring (serial fecal markers, growth, and endoscopic reassessment when indicated).

Barrier, oral-phase dysfunction, and digestion as amplifiers of mucosal stress

A clinically distinctive element of this case was evidence of inefficient oral-phase processing (rapid swallowing, limited chewing, texture aversion, and visible food fragments in stool) alongside fecal findings consistent with incomplete digestion. Oral processing is not simply behavioral: mastication and saliva contribute to bolus formation, lubrication, and the earliest phase of enzymatic digestion, shaping downstream gastric processing and nutrient bioaccessibility. Established physiology literature supports that chewing efficiency and salivary function can measurably alter bolus properties and early digestion dynamics.

Mechanistically, bolus particle size and oral processing patterns can influence how substrates are presented to digestive enzymes and, downstream, to the gut microbiome. Experimental and translational work indicates that differences in chewing can shift bolus particle size distributions and alter in vitro digestion kinetics; more broadly, food-derived particles that escape host digestion can persist into the lower gut and serve as microbial scaffolds or substrates [7]. While direct causal links to IBD activity are not established, this literature supports a biologically plausible pathway through which oral-phase inefficiency could increase delivery of partially processed macromolecules and fermentable structures to an already inflamed or barrier-compromised intestine [7].

The identification of a restrictive lingual frenulum with surgical correction (Figure 3) provides an anatomic correlate for limited tongue mobility and inefficient mastication/bolus formation. Importantly, contemporary pediatric guidance emphasizes that evidence for frenotomy is strongest in infant breastfeeding contexts and benefits outside that setting are less certain; therefore, in an older child the frenulum finding should be presented conservatively as a contributor to oral-motor mechanics rather than as an explanation for mucosal inflammation. In the narrative of this case, the more defensible scientific claim is that addressing oral-motor constraints and feeding therapy reduced a potentially modifiable luminal “mechanical and antigen-processing” stressor while intestinal inflammation was being monitored objectively [9].

Barrier biomarkers require similar caution in interpretation. Markers such as zonulin have been widely used in research settings, but multiple analyses highlight limitations of commercial assays and imperfect correlation with functional permeability testing; thus, barrier-related biomarkers should be framed as adjunctive signals rather than definitive measures of permeability. In this case, persistent partial abnormalities in barrier/immune markers despite symptomatic improvement can be described neutrally as consistent with the concept that epithelial and immune homeostasis may lag behind clinical remission, supporting continued longitudinal surveillance rather than mechanistic certainty.

Remission without immunosuppressants under a multimodal intestinal strategy

A central outcome in this report is improvement across multiple assessment layers, symptoms, fecal inflammatory biomarkers, and endoscopic appearance-without systemic immunosuppressive therapy. Current pediatric IBD guidance supports the use of fecal calprotectin as a valuable tool for monitoring intestinal inflammation and for correlating with endoscopic and histologic activity. Serial trends are particularly informative, and values approaching the low range increase the probability of endoscopic healing. This framing allows the case to emphasize objective monitoring (rather than symptom-only inference) when describing remission trajectory [1,2,12].

At the same time, it is important to anchor this case within standard-of-care realities. Evidence-based pediatric Crohn's disease management includes dietary induction strategies such as exclusive enteral nutrition (EEN), and many children with confirmed Crohn's disease ultimately require immunomodulatory or biologic therapy based on phenotype and risk [14]. Therefore, this report should explicitly avoid implying that immunosuppression is generally unnecessary. The scientifically appropriate claim is narrower: a carefully monitored, time-limited, gut-focused strategy may be considered in selected presentations when families decline systemic therapy, provided there is tight surveillance and a predefined threshold for escalation if inflammation persists or worsens.

The mechanistic interpretation should also remain conservative because the intervention was multimodal (dietary modification, infection/overgrowth-directed therapies, digestive/oral-phase interventions, and microbiota modulation). Pediatric IBD diagnostic criteria and guidelines emphasize excluding infectious etiologies and considering mimickers; additionally, a large body of literature supports the concept that gut microbiota-immune interactions can shape mucosal inflammation and systemic immune tone. Within that context, it is reasonable to hypothesize that reducing luminal inflammatory drivers (including infections, dysbiosis, or food-triggered immune activation) contributed to the observed improvement, but disentangling the contribution of each component is not possible from a single-case design [2,11].

Finally, the paired baseline and follow-up colonoscopy images (Figure 1) strengthen the report by documenting objective endoscopic improvement, a clinically meaningful outcome that extends beyond symptom resolution. The follow-up findings should be interpreted conservatively as evidence of mucosal healing or near-healing, without implying definitive disease reclassification or excluding the need for continued clinical surveillance. Overall, the most defensible conclusion is that this case is hypothesis-generating and supports further prospective evaluation of phenotype-informed, multimodal intestinal strategies in pediatric colitis presentations that include strong allergy/histamine features and demonstrable oral-phase dysfunction, under rigorous, guideline-aligned monitoring [12].

This report should not be interpreted as evidence against immunosuppressive or biologic therapy in pediatric Crohn's disease. Rather, it illustrates that in selected Crohn-like or IBD-spectrum presentations with allergic, infectious, histamine-related, and oral-phase features, a tightly monitored multimodal gut-focused approach may be clinically informative when families decline systemic therapy.

Strengths and limitations

Strengths of this report include longitudinal follow-up, objective inflammatory stool biomarkers at baseline and follow-up, endoscopic reassessment demonstrating mucosal improvement, detailed characterization of dietary, histamine-related, microbiota-related, and oral-phase digestive features, and inclusion of the parent perspective. These elements strengthen the clinical description beyond symptom reporting alone and support the value of objective monitoring in a complex Crohn-like pediatric colitis presentation.

This is a single-case descriptive report, and causality cannot be established. Natural symptom fluctuation cannot be excluded. The intervention was deliberately multimodal, and the independent contribution of each component cannot be disentangled. Follow-up biomarkers and colonoscopy were available, but the approach relied heavily on clinical and family-reported measures between testing time points. Observational bias is possible because treatment and assessment were not blinded. Generalizability is limited, particularly to children without allergic or histamine-related features. Finally, mechanistic conclusions about specific pathways (histamine signaling, barrier repair, or microbiota shifts) cannot be drawn from this case alone.

## Conclusions

This case describes an early-onset, Crohn-like chronic colitis phenotype in a child with an allergic history, histamine-pathway variants, barrier activation markers, and impaired mastication/digestive processing. Over approximately two years, a structured, nonimmunosuppressive, gut-focused multimodal strategy was associated with resolution of hematochezia, improvement of stool inflammatory biomarkers, and near-complete mucosal healing on repeat colonoscopy. The case is hypothesis-generating and supports further prospective study of phenotype-directed, stepwise approaches in selected pediatric chronic colitis presentations.
